# Exploring the accessibility of primary health care data in Europe's COVID-19 response: developing key indicators for managing future pandemics (Eurodata study)

**DOI:** 10.1186/s12875-024-02413-5

**Published:** 2024-06-20

**Authors:** Sara Ares-Blanco, Marina Guisado-Clavero, Charilaos Lygidakis, María Fernández-García, Davorina Petek, Shlomo Vinker, Donald Li, Anna Stadval, José Joaquín Mira Solves, Lourdes Ramos Del Rio, Ileana Gefaell Larrondo, Louise Fitzgerald, Limor Adler, Radost Assenova, Maria Bakola, Sabine Bayen, Elena Brutskaya-Stempkovskaya, Iliana-Carmen Busneag, Asja Ćosić Divjak, Maryher Delphin Peña, Philippe-Richard Domeyer, Dragan Gjorgjievski, Mila Gómez-Johansson, Miroslav Hanževački, Kathryn Hoffmann, Oкcaнa Iлькoв, Shushman Ivanna, Marijana Jandrić-Kočić, Vasilis Trifon Karathanos, Aleksandar Kirkovski, Snežana Knežević, Büsra Çimen Korkmaz, Milena Kostić, Anna Krztoń-Królewiecka, Bruno Heleno, Katarzyna Nessler, Heidrun Lingner, Liubovė Murauskienė, Ana Luisa Neves, Naldy Parodi López, Ábel Perjés, Ferdinando Petrazzuoli, Goranka Petricek, Martin Sattler, Natalija Saurek-Aleksandrovska, Bohumil Seifert, Alicia Serafini, Theresa Sentker, Paula Tiili, Péter Torzsa, Kirsi Valtonen, Bert Vaes, Gijs van Pottebergh, Raquel Gómez-Bravo, Maria Pilar Astier-Peña

**Affiliations:** 1 Federica Montseny Health Centre, Gerencia Asistencial de Atención Primaria, Servicio Madrileño de Salud, Madrid, Spain; 2 Instituto de Investigación Sanitaria Gregorio Marañón, Madrid, Spain; 3 SemFYC representative in EGPRN (European General Practitioner Research Network), Madrid, Spain; 4 Investigation Support Multidisciplinary Unit for Primary Health Care and Community North Area of Madrid, Madrid, Spain; 5 World Organization of Family Doctors (WONCA) Chief Executive Officer, Brussels, Belgium; 6 Las Cortes Health Centre, Gerencia Asistencial de Atención Primaria, Servicio Madrileño de Salud, Madrid, Spain; 7 semFYC Vice-Chair, Madrid, Spain; 8 Department of Family Medicine, Faculty of Medicine, University of Ljubljana, Ljubljana, Slovenia; 9 EGPRN, Brussels, Belgium; 10 Department of Family Medicine, Faculty of Medicine, Tel Aviv University and WONCA Europe President, Tel Aviv, Israel; 11 World Organization of Family Doctors (WONCA) Past president, Brussels, Belgium; 12 World Organization of Family Doctors (WONCA) President, Brussels, Belgium; 13 Universidad Miguel Hernández, Elche, Spain; 14 Fundación de Investigación e Innovación Biosanitaria de Atención Primaria (FIIBAP), Madrid, Spain; 15 Red de Investigación de Cronicidad, Atención Primaria y Promoción de la Salud (RICAPPS), Barcelona, Spain; 16 Member of Irish College of General Practice (MICGP), Member of Royal College of Physician (MRCSI), Dublin, Ireland; 17 Department of Family Medicine, Sackler Faculty of Medicine, Tel Aviv University, Tel Aviv, Israel; 18 Department Urology and General Practice, Faculty of Medicine, Medical University of Plovdiv, Plovdiv, Bulgaria; 19 Research Unit for General Medicine and Primary Health Care, Faculty of Medicine, School of Health Science, University of Ioannina, Ioannina, Greece; 20 Department of General Practice, University of Lille, Lille, France; 21 General Medicine Department, Belarusian State Medical University, Minsk, Belarus; 22 Spiru Haret” University, Occupational Health Expert, Practicing family doctor, Bucharest, Romania; 23 Health Centre Zagreb West and Department of Family Medicine, University of Zagreb, Zagreb, Croatia; 24 Department of Geriatric Medicine, Hôpitaux Robert Schuman, Luxembourg, Luxembourg; 25 School of Social Sciences, Hellenic Open University, Patra, Greece; 26 Center for family medicine, Medical faculty Skopje, Skopje, North Macedonia; 27 Capio Kvillebäcken Health Centre, Gothenburg, Sweden; 28 General Practice and Primary Care, Med. University of Vienna, Vienna, Austria; 29 Department of Family Medicine and Outpatient Care, Medical Faculty 2, Uzhhorod National University, Uzhhorod, Ukraine; 30 Health center Krupa na Uni, Republic of Srpska, Bosanska Kupra, Bosnia and Herzegovina; 31 Medical Department, Medical Education Unit, Laboratory of Hygiene and Epidemiology, Faculty of Health Sciences, University of Ioannina- Greece. GHS, Larnaca, Cyprus; 32 Faculty of Medicine, Ss. Cyril and Methodius University, Skopje, North Macedonia; 33 Health center Kraljevo, Kraljevo, Serbia; 34 Van Gürpınar District Public Hospital, Istambul, Turkey; 35 Dr Đorđe Kovačević Health Center, Lazarevac, Belgrade, Serbia; 36 Department of Family Medicine, Andrzej Frycz Modrzewski Krakow University, Krakow, Poland; 37 Comprehensive Health Research Center, NOVA Medical School, Universidade Nova de Lisboa, Lisbon, Portugal; 38 USF das Conchas, Regional Health Administration Lisbon and Tagus Valley, Lisbon, Portugal; 39 Department of Family Medicine, UJCM at Uniwersytet Jagielloński - Collegium Medicum, Krakow, Poland; 40 Hannover Medical School, Center for Public Health and Healthcare, Hannover, OE Germany; 41 Department of Public Health, Institute of Health Sciences, Faculty of Medicine, Vilnius University, Vilnus, Lithuania; 42 Imperial College London, London, UK; 43 Faculty of Medicine, University of Porto, Porto, Portugal; 44 Närhälsan Kungshöjd Health Centre, Gothenburg, Sweden; 45 Department of Clinical Pharmacology, Sahlgrenska University Hospital, Gothenburg, Sweden; 46 Department of Family Medicine at the University of Semmelweis, Budapest, Hungary; 47 Department of Clinical Sciences in Malmö, Centre for Primary Health Care Research, Lund University, Malmö, Sweden; 48 European Parliament, Luxembourg, Luxembourg; 49 PZU Femilihelt, Skopje, North Macedonia; 50 Charles University, First Faculty of Medicine, Institute of General Practice, Prague, Czech Republic; 51 Azienda Unità Sanitaria Locale di Modena, Laboratorio EduCare, University of Modena and Reggio Emilia, Modena, Italy; 52 Center for Public Health and Healthcare, Hannover Medical School, Hannover, Germany; 53 Communicable Diseases and Infection Control Unit, City of Vantaa, Vantaa and University of Helsinki, Helsinki, Finland; 54 Department of Family Medicine, Semmelweis University, Budapest, Hungary; 55 Department of Public Health and Primary Care, KU Leuven, Leuven, Belgium; 56 Academisch Centrum voor Huisartsgeneeskunde KU Leuven Kapucijnenvoer, Leuven, Belgium; 57 CHNP, Rehaklinik, Ettelbruck, Luxembourg; 58 Department of Behavioural and Cognitive Sciences, Research Group Self-Regulation and Health, Institute for Health and Behaviour, Faculty of Humanities, Education, and Social Sciences, Luxembourg University, WONCA SIGFV Executive, SSLMG Executive, Luxembourg, Luxembourg; 59 Universitas Health Centre, Public Health Service of Aragon, Zaragoza, Spain; 60 Chair of Patient Safety Working Group of Semfyc (Spanish Society for Family and Community Medicine) and and SECA (Spanish Society for Healthcare Quality) Board Member, Madrid, Spain

**Keywords:** COVID-19, Epidemiological monitoring, Primary health care, Health information systems, Europe, Health system plan

## Abstract

**Background:**

Primary Health Care (PHC) plays a crucial role in managing the COVID-19 pandemic, with only 8% of cases requiring hospitalization. However, PHC COVID-19 data often goes unnoticed on European government dashboards and in media discussions. This project aims to examine official information on PHC patient care during the COVID-19 pandemic in Europe, with specific objectives: (1) Describe PHC’s clinical pathways for acute COVID-19 cases, including long-term care facilities, (2) Describe PHC COVID-19 pandemic indicators, (3) Develop COVID-19 PHC activity indicators, (4) Explain PHC’s role in vaccination strategies, and (5) Create a PHC contingency plan for future pandemics.

**Methods:**

A mixed-method study will employ two online questionnaires to gather retrospective PHC data on COVID-19 management and PHC involvement in vaccination strategies. Validation will occur through focus group discussions with medical and public health (PH) experts. A two-wave Delphi survey will establish a European PHC indicators dashboard for future pandemics. Additionally, a coordinated health system action plan involving PHC, secondary care, and PH will be devised to address future pandemic scenarios. Analysis: Quantitative data will be analysed using STATA v16.0 for descriptive and multivariate analyses. Qualitative data will be collected through peer-reviewed questionnaires and content analysis of focus group discussions. A Delphi survey and multiple focus groups will be employed to achieve consensus on PHC indicators and a common European health system response plan for future pandemics. The Eurodata research group involving researchers from 28 European countries support the development.

**Discussion:**

While PHC manages most COVID-19 acute cases, data remains limited in many European countries. This study collects data from numerous countries, offering a comprehensive perspective on PHC’s role during the pandemic in Europe. It pioneers the development of a PHC dashboard and health system plan for pandemics in Europe. These results may prove invaluable in future pandemics. However, data may have biases due to key informants’ involvement and may not fully represent all European GP practices. PHC has a significant role in the management of the COVID-19 pandemic, as most of the cases are mild or moderate and only 8% needed hospitalization. However, PHC COVID-19 activity data is invisible on governments’ daily dashboards in Europe, often overlooked in media and public debates.

**Supplementary Information:**

The online version contains supplementary material available at 10.1186/s12875-024-02413-5.

## Introduction

The COVID-19 pandemic, with over 661 million confirmed cases as of January 2023, predominantly impacts mild and moderate cases handled within primary health care (PHC) settings. Only 8% of reported cases necessitated hospitalization, a tendency diminishing as vaccination efforts progress with the active participation of PHC professionals [1]. Despite the pivotal role played by PHC, it remains overlooked on government dashboards and in media discussions.

### Current COVID-19 pandemic information

Public health (PH) agencies worldwide furnish data on COVID-19, focusing on reported cases, testing, hospital occupancy, and vaccination. However, none spotlight the pandemic’s impact on PHC, underscoring a critical information gap [2–4]. The pandemic has reshaped healthcare delivery, reducing face-to-face appointments while increasing remote consultations, particularly for mild and moderate cases [5–7]. New clinical pathways for COVID-19 cases were established, with RT-PCR testing often conducted in PHC [8]. Limited availability of PHC open data globally, mainly from countries with public provision health systems and population-based PHC information systems [9–11], or public facilities within predominantly private provision systems [12], underscores the necessity for standardized reporting, especially in private healthcare systems [13].

### Interprofessional collaboration during the COVID-19 pandemic to guarantee comprehensive care

An effective pandemic response requires collaboration among PHC. The integration of health information is crucial, emphasizing the necessity to include PHC in pandemic dashboards and provide comprehensive training for PHC professionals. It is well-acknowledged that both PHC and PH are essential services, with a shared goal of promoting the health of the global community. However, their roles are complementary. For instance, PHC performs certain PH functions such as screening, immunization, and interventions to support healthy lifestyles, while PH enhances the effectiveness of PHC by addressing issues like health and disease surveillance, planning, and evaluation [14].

Historically, pandemics lacked PHC data, with no registered data from PHC for previous events like the SARS, MERS, H1N1 influenza, Zika, and Ebola pandemics [15]. However, information from PHC has been provided for other PH issues, such as influenza [16, 17], and various health conditions [18, 19]. Desborough et al. [15] proposed recommendations for enhancing the COVID-19 pandemic response from PHC. These suggestions included improving collaboration, communication, and integration between PH and PHC, defining the role of PHC during pandemics to offer consistent, coordinated, and reliable information from a common, trusted PHC source, involving PHC experts in national health crisis commissions, and ensuring the ability to evaluate intervention effectiveness. It is evident that training PHC professionals in their PH role could contribute to enhancing this interoperability.

### Factors affecting the lack of PHC open data during the COVID-19 pandemic

Nevertheless, collecting PHC data presents certain challenges, such as code variability, misclassification [20], or the use of free text to record information related to essential clinical data for epidemiological surveillance [21, 22]. Moreover, the interoperability of electronic health records (EHR) among different health system levels (PH, PHC and seconday care) is exceptionally uncommon among providers, regions within a country, and internationally [23]. A contributing factor to the absence of publicly available European PHC data may be linked to the lack of interoperability between PHC information systems and PH administration information systems within European countries [24]. It is noteworthy that there is no agreed-upon minimum set of patient health data for national and European PHC, and consequently, a collection of pertinent epidemiological elements that could be centralized [25]. This stands in contrast to the approach taken with the COVID-19 vaccination certificate across Europe (Green Card). Table 1 outlines various reasons for the absence of open PHC data availability, addressing and explaining each contributing factor.

**Table 1:** Identification of areas for improvement in information technologies systems in primary health care during the COVID-19 pandemic

Information Technology (IT) systems requirements for decision-making in Primary Health Care (PHC) in COVID-19 pandemic	
**Areas of improvement**	**Potential solutions**
**Data Representativeness**	
Ensuring comprehensive community-level information for effective pandemic tracking.	Unique Citizen identification: For tracing, vaccination, and medical service usage, (including vulnerable patients, low socioeconomic status, undocumented migrants, etc.) ensuring privacy through anonymization prior to data sharing.
**Information sharing between levels of care**	
Communication among the Healthcare Professionals (HCP) involved in patient care, independently of their level of care	Integrated Electronic Health Records (EHR: Shared across PHC, public health (PH), and secondary care levels, encompassing key epidemiological data. To share a common minimum epidemiological patient data set. This should include standardized common data regarding sociodemographic data, diagnosis tests, contacts tracing, consultations at the health system, other clinically relevant information from PHC, PH, A&E, hospitalization, and follow-up after the acute phase.
Communication between health insurances and PH	Obtaining data from the electronic invoicing to get more detailed information regarding the COVID-19 activity.
COVID-19 Apps	Enhanced COVID-19 Tracking through Apps: Linking COVID-19 apps with PHC and PH for a comprehensive dataset.
**Clinical Information**	
High variability on classification and coding medical care provided in PHC	Unified Medical Coding System: Standardizing terminology for effective data collection and interoperability in PHC. The coding systems used in electronic medical record systems in PHC should be unified in a patient-level coded information. Pursuit of a common classification of Diseases among Healthcare providers (Currently: International Classification of Diseases (ICD) coding (ICD-9, ICD-10) and International Classification of PHC coding (ICPC, ICPC-2)) among HCP.
Detect misdiagnosis and lack of coding during the consultation	Improving medical coding education with standard training
Suspicious cases of COVID-19 coding that are not recoded after confirmation of COVID-19 diagnosis	Automated Coding in Medical Records: introducing automatic coding updates.
**IT systems reliability and temporality**	
To guarantee the quality of the data	Data Quality and Validation: Implementing quality checks and semantic analysis for data accuracy.
Data collection and processing	Comprehensive Health System Repository: Facilitating data comparison at various level: databases, outcomes, patient-level, and population-level data (regional, national and international).
Temporality	Regular data updates allowing large-scale and real-time analysis
Open data	Open Access Dashboard: Providing clinicians, researchers, and policymakers with easy access to data for monitoring and strategic planning.
Workforce	Professional Teams for Data Analysis: Establishing dedicated teams for analyzing PHC data.
** Legislation**	Legislation for Interoperability: Mandating common data standards, particularly a minimum common set of clinical-epidemiological personal data across health information systems (PH, PHC and secondary care), for improved quality patient care and follow up of Pandemics.
** Funding Sources**	Funding Prioritization: Emphasizing investment in health IT systems within healthcare funding.

**Fig. 1:**
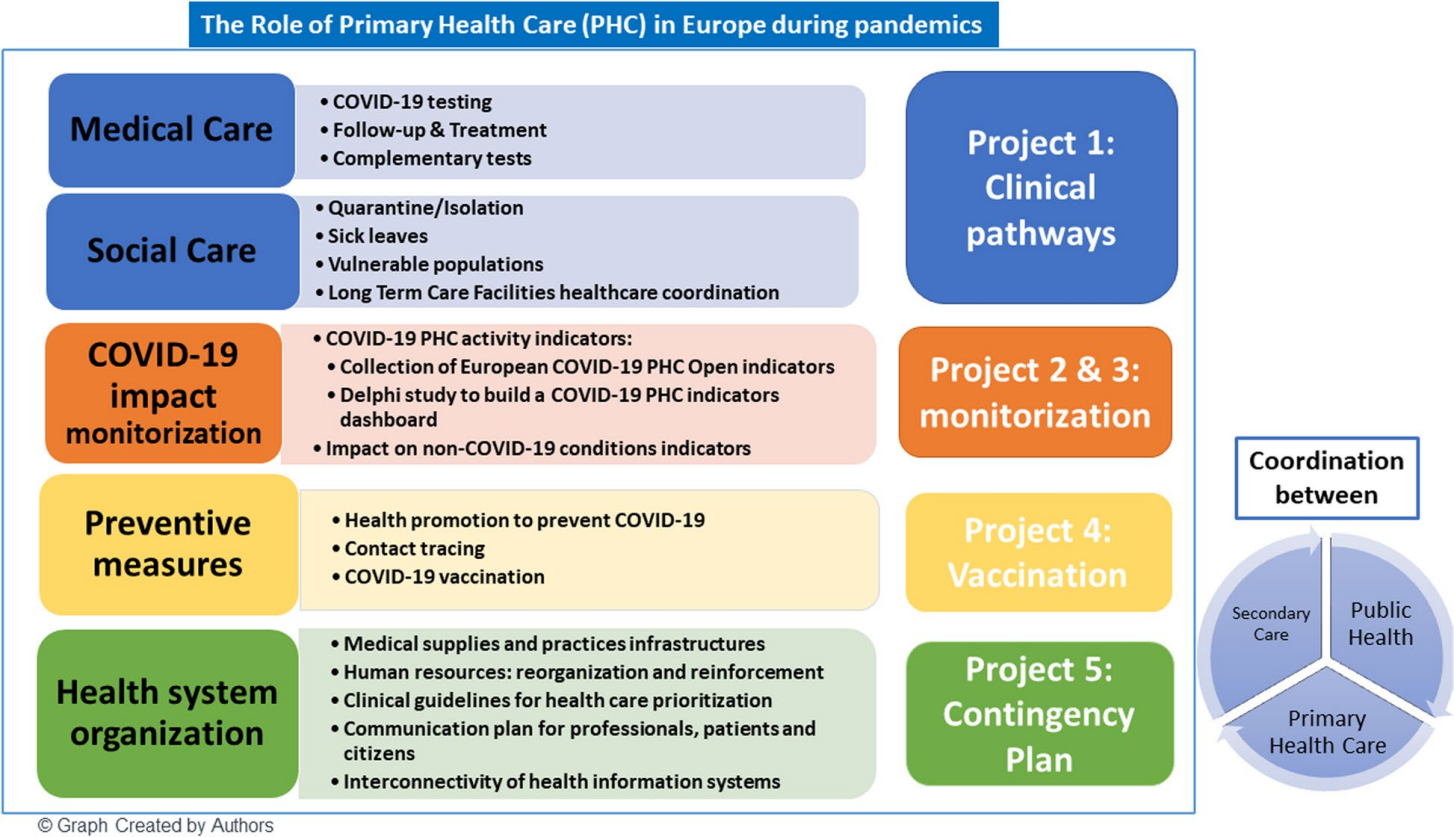
The role of primary health care in Europe during pandemics PHC: Primary health care

 A centralized data repository containing pertinent clinical and epidemiological information about COVID-19 management could have facilitated the integration of PHC COVID-19 activity indicators with data from other departments like microbiology labs and accident and emergency departments. The mentioned set of health information from patients’ EHR is relevant to have timely insights into the evolution of the pandemic. Moreover, it would not only enhance information for integrated care but also contribute to scientific research and healthcare planning, as illustrated in Fig. 1. Furthermore, there is currently a lack of cross border interoperability and secure access to EHR. The European Commission has already issued recommendations on this topic and is currently working on a legislative proposal on a European Health Data Space [26]. This initiative aims to facilitate data access and sharing among countries, addressing challenges such as interoperability of health information systems.

In many countries with a comprehensive PHC network, integrated health information systems exist, which could enable the incorporation of PHC data into the national COVID-19 dashboard [27]. Including COVID-19 PHC data would contribute to establishing an expanded pandemic dashboard in leading institutions and agencies (WHO, CDC, ECDC, etc.). New initiatives, such as the National PHC Data Collection of the Australian government, are building new health information systems that involve all stakeholders, including PHC [28]. However, it is surprising that recent legislation like the European Union’s (EU) recent proposal for the ECDC regulation, fails to mention standardized PHC data collection [29].

### Proactive planning for future health challenges: strengthening PHC systems for crisis response

During the COVID-19 pandemic, numerous digital health tools emerged, becoming an immediate necessity, and their usage significantly increased [30]. COVID-19 apps were developed in most of European countries without integration with PHC information systems.

Consequently, the full potential of these apps was not appropriately harnessed due to a lack of healthcare continuity.

This transformative approach necessitates significant funding for national and international PHC health information systems, particularly in Europe, to effectively address future health challenges. WHO Europe is urging all countries to allocate an additional 1% of the gross domestic product to PHC after the pandemic [31]. An in-depth policy analysis and interviews with family physicians across Europe could contribute to the establishment of a reliable European health information system, similar to successful initiatives in other countries [32].

The EU4Health program and other initiatives [33, 34] should allocate targeted funds to strengthen PHC, ensuring a comprehensive perspective on healthcare system performance. Some European initiative refers to research data base as the European Health Information Portal [35]. It contains catalogues for data sources, national and European projects, research infrastructures, capacity building activities, and COVID-19 related resources. This portal aids researchers in finding and accessing population health information promptly. This project originated from the Joint Action on Health Information InfAct (Information for Action! ) was funded by the European Commission, involving 40 partners in 28 EU and associated countries [36]. Presently, policymakers and healthcare system managers continue to primarily base their decisions on data from hospitals, mortality, and vaccination records.

The aftermath of the COVID-19 pandemic offers a valuable opportunity to enhance the utilization of digital health tools, with a particular emphasis on integrating PHC data. This effort should also prioritize making knowledge accessible, including within PHC, which serves as the initial point of contact for population healthcare [37]. This includes research on the development of PHC dashboards based on EHR [38]. It is now more urgent than ever to provide a comprehensive view of healthcare system performance in the context of the COVID-19 pandemic.

The assessment of the ongoing pandemic presents a crucial opportunity to elevate the use of digital health tools based on PHC data, addressing new health challenges efficiently and on a population basis. This approach will enhance the interoperability of EHR with PH and secondary care, ultimately leading to the establishment of national and European open population-based PHC data.

## Methods

The study aims to gather information on PHC, specifically focusing on medical and social care, the impact of COVID-19, monitoring, prevention, and response plans for future pandemics within the health system, as illustrated in Fig. 1. To operationalize these objectives, we have implemented five distinct research projects (Fig. 1):

- *Project 1*: Description of PHC clinical pathways regarding COVID-19 acute cases in European countries and the role of PHC in long-term care facilities (LTCF) COVID-19 clinical pathways in two different pandemic momentums: first wave and after vaccination roll out.
- *Project 2*: Description of current COVID-19 pandemic public PHC indicators available in Europe.
- *Project 3*: Elaboration of a PHC indicators dashboard regarding COVID-19 pandemic in Europe.
- *Project 4*: Description of PHC role in COVID-19 vaccination strategy roll out in European countries.
- *Project 5*: To create a comprehensive health system plan to efficiently address a pandemic scenario in PHC in Europe.

### Projects’ design

The following information is accordingly with STROBE checklist [39]. A summary of the design of all the projects can be found in Table 2.

**Table 2:** Summary of the methodology of the projects

**Methodology**			
Projects	**Questionnaire**	**Qualitative study**	**Delphi study**
1: Description of PHC clinical pathways	X		
2: Description of current COVID-19 pandemic public PHC indicators	X		
3: Elaboration of a PHC indicators dashboard		X	X
4: Description of PHC role in COVID-19 vaccination strategy roll out	X		
5: To create a contingency plan		X	X

*PHC* Primary Health Care

#### Design Project 1, 2 and 4

A mixed-methods descriptive study will be carried out, through two online self-administrated ad hoc questionnaire. The questionnaires will be built on information from official sources and consensus will be achieved among all key informants. The first to collect retrospective data on the management of COVID-19 cases, COVID-19 indicators, and COVID-19 vaccination in PHC (Annex 2). Then a flow diagram of acute COVID-19 cases, PHC highlighting strengths and weaknesses will be set as of September 2020 and April 2021, to compare pre and post vaccination clinical pathways. The LTCF healthcare will also be included. The second questionnaire will be prepared to collect detailed information on the role of PHC in national vaccination strategies in Europe.

### Participants

#### Structure of the research consortium

The research consortium is organized with a core team consisting of four specialists in family and community medicine, including one with expertise in PH. These professionals are affiliated with the Spanish Society for Family and Community Medicine (semFYC) [40] and are primarily based in Spain. The core team serves as the central coordinating body for a broader European research initiative.

Within the European research team, the role of country lead researchers is pivotal as national key informants. These lead researchers, who are healthcare professionals representing various European countries, are often affiliated with the World Organization of Family Doctors in Europe (WONCA Europe) [41]. WONCA Europe, boasting a membership of 47 organizations and a network of over 90,000 family doctors across Europe, includes semFYC as its Spanish affiliate (see Annex 1). Both WONCA Europe and semFYC actively support professional development, research, education, and quality enhancement in general practice and family medicine through various networks and specialized interest groups.

A significant number of the country’s lead researchers are members of the European General Practice Research Network (EGPRN). EGPRN, a collaborative working group comprising professionals from PHC and various disciplines, is dedicated to advancing medical research in this field [39–42]. Serving as a dynamic platform, EGPRN facilitates collaboration among researchers from diverse European countries, fostering joint research efforts in PHC.

### Study participants

The participants are key informants from each country, both from the field of PHC and PH for all the studies. For Projects 1, 2 and 4, the participants will be part of the group of collaborators mentioned in Annex 1. For the Projects 3 and 5, the professionals will be recruited by the national collaborators and the core group.

#### Data collection projects 1, 2 and 4

##### Variables and analysis (Projects 1 and 2)

Before commencing the study, national collaborators will receive invitations to attend informative webinars conducted by the research core team. Additionally, a comprehensive project overview will be communicated to them via email. Individuals expressing willingness to participate will be designated as national key informants. To formalize their participation, all key informants will sign an informed consent form. Subsequently, they will be emailed the two questionnaires for completion (see Annex 2, 3, 4).To ensure a timely response, two reminders will be sent, and once completed, the questionnaires will be closed. All information gathered through the questionnaires will be meticulously managed using a database for subsequent analysis.

A descriptive analysis of the categorical variables will be performed. Data will be displayed with the absolute number observed and the frequency (percentage). In the case of quantitative variables, these will be presented in means and standard deviations, or median and interquartile range, depending on their type of distribution. The differences among sex, age group and occupation will be tested by use of T-Student test for independent data or an ANOVA test.

##### Design projects 3 and 5

To respond to the research objectives, Projects 3 and 5 will be structured as follows.

(i) Focus groups composed by family medicine and PH specialists from different European countries to address the possible PHC indicators and the key areas to perform a PHC contingency plan for pandemics in Europe. Participants were recruited through professional networks linked to WONCA EUROPE and academic networks.
(ii) Development of a two-round Delphi study for the development of PHC activity indicators for the COVID-19 pandemic (Common PHC Pandemic Dashboard).
(iii) Elaboration by consensus of a coordinated response health system plan focused on PHC and PH to face future pandemic scenarios (contingency plan).

#### Data collection projects 3 and 5

The interviews and meetings will be recorded for the subsequent acquisition of the information that emerged in the focus groups. All the information will be collected in English. Data will be collected in the first 18 months of the pandemic since March 2020. Vaccination data will be collected during the first year of the roll up of the vaccination. A content analysis will be performed to get consensus on crucial items.

##### Variables and analysis projects 3 and 5

- Sociodemographic variables: gender (man, woman, other), age range (18–45, 46–65, > 66 years), occupation (Family Doctor, PH, other medical specialist), health field (public/private),
- Countries of the EU and origin of the information obtained: Austria, Belgium, Bulgaria, Croatia, Cyprus, the Czech Republic, Denmark, Estonia, Finland, France, Germany, Greece, Hungary, Ireland, Italy, Latvia, Lithuania, Luxembourg, the Netherlands, Poland, Portugal, Romania, Spain, Slovakia, Slovenia, Sweden.
- Other European countries: categorical nominal (Iceland, Norway).
- Detailed description PHC activity indicators during COVID-19 Pandemic.

##### Qualitative methods

The focus groups and Delphi survey are revised by SRQR [43] checklist.

##### Focus groups

Three discussion focus groups with the key informants will hold with semi-structured questions in relation to the main constructs related to the collection of PHC data in the pandemic. Proposals for improvement that key informants deem appropriate and viable will be discussed. An interview guide will be provided to discuss the findings from previous works.

Two researchers will carry out a content analysis of the answers and will build a hierarchy of core information.

##### Delphi Study

Subsequently, based on the information collected from the survey and focus groups, researchers will design a Delphi study. Delphi will consist of a proposed list of indicators for monitoring a pandemic in PHC. The indicators will be validated through two sequential questionnaires arriving at the consensual selection of a set of indicators that will conform a dashboard. The details of the Delphi study can be found in Annex 3. The selection of indicators will be made by quartiles. Those under first quartile will be dropped out, those above the third quartile will be included, and between first and third quartile will be sent to second round [44].

- Stratification of scorecard results: A simulation of different thresholds of the indicators and the possible decisions to reinforce the care and resources at the PHC level will be carried out depending on the evolution of the pandemic.
- Statistical analysis Projects 3 and 5: A descriptive analysis of the categorical variables will be carried out, being shown with the absolute number observed and the frequency (percentage). In the case of quantitative variables, these will be expressed with the mean and standard deviation, or median and interquartile range, depending on their distribution. In the first round, participants will be asked to rate the relevance of the indicators using a 5-point Likert scale. Consensus is defined when ≥ 80% of the participants rated a statement as ‘agreed’ or ‘strongly agreed’. Those indicators which have less than 25% of acceptance were eliminated before advancing to the second round. The final indicators will be those which reach an agreement over 80%.

The data for the five projects are stored on the servers of the Madrid Health System, where the principal team members are based. The Delphi Study is carried out using the platform provided by the University Miguel Hernandez de Elche in Spain, where one of the team’s researchers is stationed. For data processing and statistical analysis, we employ STATA software, version 16.

## Discussion

The projects face potential limitations, primarily stemming from the scarcity of open available PHC data in each country. Furthermore, the representation of each country is reliant on volunteers from PHC organizations that belong to WONCA Europe, and EGPRN, which could introduce selection bias. Nevertheless, the use of publicly available data in certain projects (1, 2 and 4 ) and oversight by experienced professionals, previously involved, in others aims (3 and 5) to ensure a more uniform understanding and analysis.

Addressing the challenge of scarce PHC data publicly available, our approach includes surveying official websites for initial data gathering and directly requesting data from EU and OECD health departments when necessary. This effort is underscored by the goal of thoroughly evaluating the specified variables in our protocol.

To counter the issue of non-representative countries, we will proactively engage with their health departments for data acquisition. Our contingency plan for limited cooperation involves extensive dissemination of the project and volunteer recruitment via scientific societies and their networks (e.g., EGPRN, WONCA Europe, semFYC, etc.).

The outcomes of this study will be disseminated through diverse channels, including presentation at conferences and scientific gatherings, publication in high-impact journals, and delivery to policymakers and healthcare system administrators. We also plan to share our findings with the public and traditional media to increase awareness of the crucial role of PHC in the healthcare system. As we evaluate the current pandemic through our five projects, we see a significant opportunity to explore the efficient use of digital health tools based on PHC data, addressing future health challenges at a population level. The results may provide insights to enhance the interoperability of EHR with PHC and secondary care across the European Region, ultimately contributing to the development of a national and European open population-based PHC database.

### Supplementary Information

Supplementary Material 1.

## Data Availability

All data generated or analysed during this study can be shared upon request. All the original data can be checked directly through the Supplement files. All methods were carried out in accordance with relevant guidelines and regulations. Further inquiries can be directed to the corresponding author.

## Abbreviations

CDC: Centre for Disease Control of USA
ECDC: European Centre for Disease Prevention and Control
EGPRN: European General Practice Research Network
GP: General Practitioner or Family Doctor
NHS UK: National Health Services, United Kingdom
OECD: Organisation for Economic Co-operation and Development
PH: Public Health
PHC: Primary Health Care
RT-PCR: Real-Time - Polymerasa Chain Reaction
semFYC: Sociedad Española de Medicina Familiar y Comunitaria
WHO: World Health Organization
WONCA: World Organization of National Colleges, Academies and Academic Associations of General Practitioners/Family Physicians
