# Supplementary material for: Exploring the accessibility of primary health care data in Europe's COVID-19 response: developing key indicators for managing future pandemics (Eurodata study)
Source: BMC Prim Care. 2024 Jun 20;25:221. doi: 10.1186/s12875-024-02413-5 (PMC11188206; doi:10.1186/s12875-024-02413-5)
Supplement: Supplementary file 1 — Supplementary Material 1. [file 12875_2024_2413_MOESM1_ESM.docx]

**Supplement 1: Characteristics of WONCA Europe.** WONCA Europe is the European Regional Branch of WONCA. It represents 47 member organizations and more than 90,000 family doctors in Europe. WONCA is the academic and scientific society for general practice/family medicine across the world. Information about each national member organization is available at: https://www.woncaeurope.org/page/member-organisations

| **Country** | **Name of the organization** |
| --- | --- |
| Armenia | Armenian Association of Family Physicians |
| Austria | Austrian Association of Family Medicine |
| Belgium | Belgian Society for General Practitioners (BSGP) |
| Bosnia and Herzegovina | Association Of Family Doctors Of Republic Of Srpska and Family Medicine Association, FB&H |
| Bulgaria | BGPSRE |
| Croatia | KoHOM and HUOM |
| Czech Republic | Czech Society of GP (CSGP) |
| Denmark | DSAM |
| Estonia | The Estonian Society of Family Doctors (ESFD) |
| Finland | Finnish Asociation for General Practice (SYLY) |
| France | College of general practice (CMG) |
| Georgia | Georgia Family Medicine Association ( GFMA) |
| Germany | German Society of General Practice/Family Medicine (DEGAM) |
| Greece | ELEGEIA, Greek College of General Practitioners (GCGP), Greek College of General Practitioners (GCGP) |
| Hungary | Hungarian Research Association of Family Physicians - CSAKOSZ |
| Ireland | ICGP |
| Iceland | Icelandic College of Family Physicians |
| Israel | Israel Association of Family Physicians |
| Italy | Centro Studi e Ricerche in Medicina Generale (CSeRMEG) and Italian Academy of Family Physicians (AIMEF) |
| Kazakhstan | Kazakhstan Association of Family Physicians (KAFP) |
| Kosovo | Association of Family Physicians of Kosova (AFPK) |
| Kyrgystan | Association of Family Group Practices (FGPA) |
| Latvia | Rural Family Doctors Association of Latvia |
| Lithuania | Lituanian College of Family Physicians |
| Luxembourg | Luxemburgish Scientific Society for General Practice (SSLMG) |
| Malta | Malta College of Family Doctors |
| North Macedonia | Association of General Practice/Family Medicine Macedonia (ZLOM SM) |
| Netherlands | Dutch College of General Practitioners ( DCGP/NHG) |
| Norway | Norwegian College of General Practice (NFA) |
| Poland | The College of Family Physicians in Poland |
| Portugal | Portuguese Association of General Practice and Family Medicine ( APMGF) |
| Romania | ASNMF/MG- Romania |
| Russia | All Russian Fund - Association of General Practitioners of Russian Federation |
| Serbian | Serbian Medical Association/Section of General Practice |
| Slovakia | The Slovak Society of General Practice of the Slovak Medical Association (SkSGP/SSVPL) |
| Slovenia | Association of Slovene Family Doctors (ZZDM) |
| Spain | semFYC |
| Sweden | SFAM - Swedish Association of General Practice |
| Switzerland | Swiss Society of General Internal Medicine (SGAIM) |
| Tajikistan | Public Organization National Association of Family Medicine Workers of Tajikistan (NAFMST) |
| Turkey | TAHUD |
| UK | RCGP |
| Ukraine | The Ukrainian Family Medicine Association (UAFM) |

**Supplement 2:** Eurodata questionnaires of the management of COVID-19 cases ( project 1), COVID-19 indicators ( project 2), and COVID-19 vaccination in PHC ( project 4)

**EURODATA QUESTIONNAIRE (PROJECT 1)**

**Definitions (following the MeSH term in PubMed):**

- **Primary care:** Care which provides integrated, accessible health care services provided by a GP or a primary care nurse in the context of family and community
- **A&E:** Accident & Emergency department or Emergency Department. Hospital department responsible for the administration and provision of immediate medical to the COVID-19 patient.
- **COVID-19 Telephone Hotline:** A direct communication system, usually telephone, established for instant contact. It is designed to provide only information about COVID-19 and assistance through trained personnel and is used for counseling and referrals.
- **Public Health:** Branch of medicine concerned with the prevention and control of COVID-19 patients on the national, regional, or municipal level
- **GP:** General Practitioner or family doctor. Doctors who are responsible for the provision of comprehensive and continuing care to every individual seeking medical care irrespective of age, sex and illness and they care for individuals in the context of their community (Wonca Europe definition, <https://bit.ly/3D5HKfl>)
- **Primary care nurse:** Nurses who provide care to patients of all age levels, and who focus their efforts on the health care needs of the entire family in the context of the community.
- **Public Health doctor:** Doctor whose goal is to improve health and quality of life in a population or community through the prevention and treatment of diseases, the surveillance of cases and health indicators, and the promotion of healthy behaviors through public education and awareness.
- **Public Health nurses**: Nurses whose goal is to improve health and quality of life in a population or community through the prevention and treatment of diseases, the surveillance of cases and health indicators, and the promotion of healthy behaviors through public education and awareness.
- **Social Services:** The use of community resources, individual case work, or group work to promote the adaptive capacities of individuals in relation to their social and economic environments.

|  | **Clinical pathway of adult COVID-19 patients in General Practice (SEPTEMBER 2020)** | |
| --- | --- | --- |
| **SARS-CoV-2 testing** | If the patient has COVID-19 symptoms, who should he call to ask for medical care? * |  |
|  | Which departments or institutions are in charge of RT-PCR? * |  |
|  | Which departments or institutions are in charge of antigenic tests? * |  |
|  | Where can patients get COVID-19 testing? * |  |
|  | Is RT-PCR free of charge?# |  |
|  | Is antigenic test free of charge?# |  |
|  | Can patients get a RT-PCR on their own without a prescription?# |  |
|  | Can patients get an antigenic test without a prescription?# |  |
|  | Who is testing patients who are immobile (elderly, bed ridden)?* |  |
|  | Who is interpreting the result of the SARS-CoV-2 testing?** |  |
|  | Who is in charge to give the test result to the patient?** |  |
|  | Other comments: |  |
| **Contact Tracing** | Which department are responsible of contact tracing? * |  |
|  | Which channels are used to inform contact persons who are vulnerable? (Elderly, deaf patients, patients who don´t use a mobile phone, etc.) |  |
|  | Other comments: |  |
| **Follow-up** | Where do patient ask the sick leave? * |  |
|  | How long shall patients stay at home? ** |  |
|  | In case, the patients don´t have a room to isolate themself, are there hotel rooms or any other resource to isolate them?** |  |
|  | Who decides the lengths of the patient confinement?** |  |
|  | Is there any community organization that helps to buy food, bring meals or provide any extra need for elderly or people without social support?** |  |
|  | Who will supervise that the patient is doing the confinement correctly?* |  |
|  | Who is giving medical care and follow-up to patient´s symptoms?** |  |
|  | If the patient needs a physical exploration, who and where is the exam taking place?*,** |  |
|  | If the patient´s condition worsens, How the patient communicate with their GP? |  |
|  | Other comments: |  |
| **Additional Testing** | If the patient needs an X-ray, who is requesting it and where will it happen?*,** |  |
|  | If the patient needs a blood test, who is requesting it and where will it happen?*,** |  |
|  | Do patients need a test at the end of the confinement to be allowed to end it? |  |
|  | Other comments: |  |
| **Moderate and severe cases** | In case of moderate or severe cases***, who is responsible of patient´s care? When are patients referred to the hospital? |  |
|  | How do patients are transferred to hospital (ambulance?) Who are responsible to order an ambulance? |  |
|  | Other comments: |  |

* Primary Care, Any Hospital Department including A&E, COVID-19 Telephone Hotline, Public Health, other (please, specify)

** GP, primary care nurse, COVID-19 Telephone Hotline, Public Health doctor, Public Health nurse, Social Services, other (please, specify)

*** Moderate or severe cases are patients who have complications that do not required ICU admission (pneumonia, pulmonary thromboembolism, etc.)

# Yes, no, other (please, explain the circumstanc

| **Additional Testing** | If the patient needs an X-ray, who is requesting it and where will it happen?*,** |  |
| --- | --- | --- |
|  | If the patient needs a blood test, who is requesting it and where will it happen?*,** |  |
|  | Do patients need a test at the end of the confinement to be allowed to end it? |  |
|  | Other comments: |  |
| **Moderate and severe cases** | In case of moderate or severe cases***, who is responsible of patient´s care? When are patients referred to the hospital? |  |
|  | How do patients are transferred to hospital (ambulance?) Who are responsible to order an ambulance? |  |
|  | Other comments: |  |

* Primary Care, Any Hospital Department including A&E, COVID-19 Telephone Hotline, Public Health, other (please, specify)

** GP, Primary Care nurse, COVID-19 Telephone Hotline, Public Health doctor, Public Health nurse, Social Services, other (please, specify)

*** Moderate or severe cases are patients who have complications that do not required ICU admission (pneumonia, pulmonary thromboembolism, etc.)

| **Country General Questions** |  |
| --- | --- |
| Country of origin (please write the country you are describing in this form) |  |
| Source of information (national guideline, regional guideline, family doctor association guideline, international guideline), please provide the links. |  |
| Was there a COVID-19 telephone hotline? # |  |
| Range of age of Primary Care patients |  |
| Describe the professionals who are working in Primary Care (GP, primary care nurse, pediatrician, midwife, social workers, physiotherapies, others, etc.) |  |
| Was there remote assessment in Primary Care (telephone or video consultation)?# |  |
| Were there face-to-face appointments to explore COVID-19 patients in Primary Care?# |  |
| Primary Health Care system provider (public, private, mixed or other) |  |
| Describe the employment status in your country (salaried with the government, self-employed with contract to health insurance fund(s) or health authority, self-employed without contract (paid by patients out-of-pocket)) |  |
| Describe how Primary Health Care professionals’ salary is calculated (payment by medical act (consultation, home visit, etc.), payment by the number of patients attended, fixed salary, others) |  |
| Did Primary Care attend patients with COVID-19 in nursing homes? # |  |
| Were there any prescription restrictions in your country to prescribe COVID-19 ambulatory treatment in Primary Care (oxygen, heparin, systemic glucocorticoids, inhaled glucocorticoids, etc.)?# |  |
| Strengths of the clinical pathway of adult COVID-19 patients in General Practice (optional answer) |  |
| Weakness of the clinical pathway of adult COVID-19 patients in General Practice (optional answer) |  |
| Other comments regarding any of these questions: |  |
| Sources: Links of the COVID-19 guidelines or official sources/bibliography that you have checked to complete this form |  |

# Yes, No, Other answer (please, explain the circumstance)

**EURODATA, questionnaire for Project 2:**

| **Project 2** |
| --- |
| **Country:** |
| **Main variable** |
| **Var 1:** Is there available information in your country about the number of COVID-19 patients who were treated in PC? ( Yes/No/Other, explain) |
|  |
| **Describe all the variables regarding PC available in your country and the number of them, each variable should be describe as qualitative and quantitative** |
| **Please use and create as many variables as you need.** |
| Example: Var 2: Number of patients who were followed-up in primary care in Spain ---> Var2_num: 1.000.000-->Link: www…. |
|  |
| **Var2:** Describe an available variable regarding PC in your country |
| **Var2_num:** number of patients regarding the variable 2 from 15th of March 2020 (12th epidemiological week) until 31st of August 2021. |
| **Link var_2:** Website where the information of variable 2 has been collected |
|  |
| **Var3:** Describe an available variable regarding PC in your country |
| **Var3_num:** number of patients regarding the variable 3 from 15th of March 2020 (12th epidemiological week) until 31st of August 2021. |
| **Link_var_3:**Website where the information of variable 3 has been collected |
|  |
| **Var4:**Describe an available variable regarding PC in your country |
| **Var4_num:** number of patients regarding the variable 4 from 15th of March 2020 (12th epidemiological week) until 31st of August 2021. |
| **Link_var_4:**Website where the information of variable 4 has been collected |
|  |
| **Var5:**Describe an available variable regarding PC in your country |
| **Var5_num:**  number of patients regarding the variable 5 from 15th of March 2020 (12th epidemiological week) until 31st of August 2021. |
| **Link_var_5:**Website where the information of variable 5 has been collected |
|  |
| **Var6:**Describe an available variable regarding PC in your country |
| **Var6_num:** number of patients regarding the variable 5 from 15th of March 2020 (12th epidemiological week) until 31st of August 2021. |
| **Link_var_6:**Website where the information of variable 6 has been collected |
|  |
| **Secondary variable: GENERAL NUMBERS** |
| **Source of information consulted:** Ministry of Health website, Governmental website, insurance website, family physician association website, another website (free text to explain where). |
|  |
| **COVID-19 as a notifiable disease**: yes, no. |
| Is there a national gubernamental COVID-19 App in your country?: Yes, no |
| **Is the COVID-19 App connected to Primary Care so patients can communicate with their GP/ PC nurse?** Yes, no |
|  |
| **Which international classification is used to codify COVID-19 in the electronic health record?** ICPC coding, ICPC-2 coding, ICD-9 coding, ICD-10 coding, other ( specify) |
|  |
| **Number of people infected by SARS-CoV-2, from 15th of March 2020 (12th epidemiological week) until 31st of August 2021** |
| **Is there information about sex ( female/male) regarding the total number of cases? ( yes/no)** |
| **Is there information about the age range regarding the total number of cases? ( yes/no)** |
| **Link_infected:** Website where the information of number of total cases has been collected |
|  |
| **Number of people infected by SARS-CoV-2 hospitalized, from 15th of March 2020 (12th epidemiological week) until 31st of August 2021** |
| **Link_hospitalized:** Website where the information of number of hospitalized patients has been collected |
|  |
| **Number of people infected by SARS-CoV-2 who have required intensive care to date(ICU), from 15th of March 2020 (12th epidemiological week) until 31st August 2021** |
| **Link_ICU:** Website where the information of number of ICUpatients has been collected |
|  |
| **Number of people infected by SARS-CoV-2 who died to date from 15th of March 2020 (12th epidemiological week) until 31st of August 2021** |
| **Link_Death:** Website where the information of number of total death has been collected |

**EURODATA, QUESTIONNAIRE FOR PROJECT 4**

|  | **Vaccination roll-up of the first dose of COVID-19 vaccine in Europe**  **(Starting in December 2020 and finishing by the end of November 2021)** | |
| --- | --- | --- |
|  | **Country:** |  |
|  | **Number of 1^st^ doses of vaccines administrated between the 1^st^ of December 2020 and 30^th^ of November 2021**  **(If your country belongs to the EU, we will download the ECDC information so you can leave this box empty).** |  |
| **National organization of the COVID-19 vaccination** | Who was in charge of the roll out of the COVID-19 vaccination? |  |
|  | How does your national health institutions ensure clear communication on the benefits, risks and importance of COVID-19 vaccines? |  |
|  | Were there any relevant experience (national leaders, private campaigns, etc.) to promote the vaccination in your country? |  |
|  | Drivers of low vaccination (A few countries described some of the drivers of low vaccination acceptance and uptake in different population groups. Many countries mentioned issues of mistrust, misinformation and low perception of risk in young people as some of the main drivers of low vaccination in different population groups.) |  |
|  | Were there any strategies and measures in place for increasing vaccine uptake and acceptance? |  |
|  | If there were any strategy, which ones (mobile and pop-up vaccination teams, social media campaigns, incentives, etc.) ? did they involved PHC? |  |
|  | Were there priority groups to start the vaccination in your country? |  |
|  | Were there some number of phases in the roll-up of the first dose? |  |
|  | Did your country had enough capacity to deliver vaccines in terms of health care workers in every phase of the roll-up of the vaccination? |  |
|  | Was the vaccination mandatory in your country? |  |
|  | In case the vaccination was mandatory in some groups, please specify which groups |  |
|  | Which vaccines were available in your country? (BioNTech and Pfizer, Moderna, AstraZeneca, Janssen, Sanofi-GSK, Novanax, Valneva, CoronaVac_Sinovac, Sputnik, etc) |  |
|  | Could patients choose which vaccine was administrated to them? |  |
|  | In case, there were priority groups, could you describe these groups? (Health care workers, Personnel in LTCFs, Social care personnel, Elderly and residents in long-term care facilities, Children, Pregnant women, adults with comorbidities, etc.) |  |
|  | How was the strategy in those who had COVID-19 in the previous year to get vaccinated? How long did they have to wait from the acute infection to be eligible to get the vaccine? |  |
|  | How the identified target groups were invited to be vaccinated (invitation letter sent, sms, public campaigns, etc.)? |  |
|  | Are health authorities and civil protection authorities coordinating in your country to deploy COVID-19 vaccines? |  |
|  | Please provide the sources to the answers in these section |  |
|  | Other comments |  |
| **General campaign** | How vaccination is delivered? Locations/sites providing vaccinations for: schools, Vaccination centres  GP practices/family doctors, Paediatric clinics, Hospital, Pharmacies |  |
|  | In the locations which are not health settings, which healthcare professionals were responsible to administer the vaccine? |  |
|  | Do health care professionals advice regarding possible side effects of the vaccine before the administration? |  |
|  | Once the vaccination is provided, how much time the patients were waiting before being discharge? |  |
|  | In case, there were a side effect of the vaccination after the administration, which healthcare professional was responsible to provide care? |  |
|  | Was there any special safety recommendation to provide vaccination to the patients? |  |
|  | How patients can get an appointment to be vaccinated? |  |
|  | Was there any special campaign to assure any vulnerable group could be vaccinated? In case yes, please describe which vulnerable group and the strategy to facilitate the access to vaccination  **We are not asking medical conditions but vulnerable population as homeless, illegal immigrants, etc. |  |
|  | Can you get the vaccination without an appointment to ensure vulnerable groups are vaccinated? |  |
|  | Some countries described a change in the initial strategy because of low vaccination rates in the first year of the vaccination roll-out. If this is the case in your country, could you explain the situation? Motives to change the strategy, main characteristics of the new strategy |  |
|  | If there were some problems/challenges in the organization of the vaccination, please explain what happened |  |
|  | Did healthcare institutions receive extra funding for running the vaccination campaign? In case the answer is affirmative, please explain how the extra funding was spent |  |
|  | Did healthcare professionals receive an extra pay for running the vaccination campaign? In case the answer is affirmative, explain which professionals received it. |  |
|  | Please provide the sources to the answers in these section |  |
|  | Other comments |  |
| **PHC Participation in the vaccination** | Which vaccines were administered in PHC settings? |  |
|  | At the beginning of the vaccination, the temperature to store the vaccines were:  *Moderna and Jansen: -20ºC  *Pfizer: -80ºC  Did the PHC settings have freezers to store vaccines? How the cold chain was organized in PHC? Did they receive vaccines from Public Health/Hospital settings every day? |  |
|  | Does PHC participate in administering vaccines to the population? In case the answer is affirmative, describe what kind of patients were vaccinated in PHC |  |
|  | Which PHC professional was participating in administering vaccines? (Nurse, GP, midwife, etc.) |  |
|  | Do PHC health professionals who administered vaccines were economically compensated to take this task? |  |
|  | How do patients get an appointment in PHC to be vaccinated? |  |
|  | Was there any special campaign to assure any vulnerable group could be vaccinated in PHC? In case yes, please describe which vulnerable group and the strategy to facilitate the access to vaccination  **We are not asking medical conditions but vulnerable population as homeless, illegal immigrants, etc. |  |
|  | Do health care professionals advice regarding possible side effects of the vaccine before the administration? |  |
|  | Once the vaccination is provided, how much time the patients were waiting before being discharged? |  |
|  | In case, there were a side effect of the vaccination after the administration, which healthcare professional was responsible to provide care? |  |
|  | Please provide the sources to the answers in these section |  |
|  | Other comments |  |
| **Information system** | Does your country have an Immunization Information System or another type of vaccination registry? |  |
|  | Who is in charge of the Immunization Information System? (Public Health, Ministry of Health, PHC, etc.) |  |
|  | Is the Immunization Information System online, paper registry? |  |
|  | Do PHC have platform to register the administered vaccines? Is it online or on paper? |  |
|  | What kind of data is recorded? (patient´s name, patient´s ID, type of vaccination, arm of administration, professional who administered it, etc.) |  |
|  | In case, there is an online platform in PHC, is this platform connected with the electronic health record of the patient in primary care? |  |
|  | Who is the person responsible to register the vaccination in the information system? |  |
|  | In case there is not an online registration system, please explain how the registration is made and which professionals are involved in the registration |  |
|  | Is there a national registration to monitor the vaccination roll-up? Is this information open data?  *Open data: Public data that anyone can access, use and share from national/regional institutions* |  |
|  | Is there information regarding the sex, range age, priority groups and comorbidities in the population who has already received the vaccination in the national registration? |  |
|  | Is there information regarding where the vaccination happened (PHC, Hospitals, other settings, etc.) in the national registration? |  |
|  | Please provide the sources to these questions in these section |  |
|  | Other comments |  |
| **Vaccination side effects** | In case of side effects of the vaccinate, how can the patient notify the side effect? (Online, phone, by paper, etc.) |  |
|  | In case of side effects, who will be the institution (emergency hotline, PHC, PH, Pharmacy, etc.) to attend the patient if he has doubts or if he is seeking medical care? |  |
|  | In case the patients are asking for medical care in PHC, does PHC notify the side effects? |  |
|  | Which PHC professional notify the side effects? |  |
|  | How is the notification done? (Online, by paper, etc.) |  |
|  | Is there a national or regional registration of vaccination side effects? |  |
|  | In case yes, is it online or on paper record? |  |
|  | In case no, is there an indirect record as the insurance data base regarding the vaccination? |  |
|  | Is this national or regional registration open data in a website?  *Open data: Public data that anyone can access, use and share from national/regional institutions* |  |
|  | In case the patient needs a sick leave for the side effects, who is responsible for the sick leave? |  |
|  | Please provide the sources to these questions in these section |  |
|  | Other comments |  |
| **COVID-19 passport** | How do you get your COVID-19 passport? Please explain the steps in your country |  |
|  | Are they mandatory to access places/events (restaurants, museums, concerts)? |  |
|  | Are they mandatory to access healthcare facilities? |  |
|  | Are they mandatory for public transport? |  |
|  | Please provide the official sources to the questions in these section |  |
|  | Other comments |  |
|  |  |  |

**Supplement 3: Delphi questionnaire**

Dear colleague:

We are a group or researchers from EGPRN (European General Practitioner Research Network), which belongs to WONCA Europe (Global Organization of Family Doctors, European Region). The EGPRN scientific committee supervised and approved the Eurodata study; they gave a small grant to cover the cost of publication.

We are conducting a research project in 30 European countries collecting data of the activity of primary care practices during COVID-19 pandemic in order to make visible the crucial role of primary care during this tough time. We found that only 13 of 30 countries collected and published indicators regarding COVID-19 in primary care. 40 different indicators have been collected but there is little consensus of which indicators are more helpful and reliable. The goal of this study is to search indicators that could be useful in the decision-making of a future pandemic contingency plan. At the same time, we would like to promote the inclusion of primary care activity data on the national and international future pandemic dashboard (Ministry of Health, ECDC, WHO, etc.) in Europe.

We are inviting you as an expert whether in family medicine and/or public health domain to participate in a Delphi study to achieve a consensus about a set of primary care COVID-19 indicators in Europe considering the perspective of GPs and Public Health officers. This would involve completing a brief online questionnaire, rating possible primary care COVID-19 indicators. It is envisaged that this should take approximately 15-20 minutes. Two Delphi rounds have been planned.

The questionnaire will be sent by an e-mail from the Universidad Miguel Hernández de Elche (Spain) and you will have two weeks to answer. If you complete all the rounds, you will be invited to be part of a collaborative authorship in an international paper and in a EGPRN conference presentation. We would be very honoured if we could count with your opinion in this study. Once finished the research study, we will send you a report on the final results. For any further information, do not hesitate to contact us.

Kind regards.

Sara Ares-Blanco [sararesb@gmail.com](mailto:sararesb@gmail.com) (ORCID: 0000-0002-4984-8788)

Raquel Gómez Bravo [raquelgomezbravo@gmail.com](mailto:raquelgomezbravo@gmail.com) (ORCID: 0000-0002-3192-7672)

María Pilar Astier-Peña [mpastier@gmail.com](mailto:mpastier@gmail.com) (ORCID: 0000-0002-3192-7672)

**SECTION 1: DEMOGRAPHIC DATA**

| QUESTION | ANSWER |
| --- | --- |
| Age | Free text |
| Sex | Male, female, other |
| Professional Background | General Practice, Public Health, other ( free text to explain) |
| Years of expertise after the specialization | <5 years, 5-9 years, 10-19 years, 20-29 years,>30 years |
| Current job | Free trext |
| Country of current practice | Free text |
| Email address | Free text |

**SECTION 2 GENERAL QUESTIONS REGARDING YOUR COUNTRY**

| Is there a sentinel Doctor network in General Practice in your country/region? | yes/no |
| --- | --- |
| Does your country have disaggregated health indicators from the PRIMARY CARE public providers/health authorities? | yes/no |
| Does your country have disaggregated health indicators from the insurance companies regarding primary care? | yes/no |
| What level of disaggregation does primary care public providers/ health authorities have at the moment? | Country level, regional level, practice level, all these levels together |
| What level of disaggregation does insurance companies have at the moment regarding primary care? | Country level, regional level, practice level, all these levels together |

**SECTION 3** **WHICH VALUES ARE RELEVANT TO CREATE AN INDICATOR IN PRIMARY CARE?**

This section looks for a consensus to the values that are more important to create an indicator for the participants. The sentences have been extracted from the WHO book “Health indicators: Conceptual and operational considerations” (2018).

| Health indicators attempt to describe and monitor a population´s health status or condition | 5-point Likert Scale:(1) Strongly disagree; (2) Disagree; (3) Neither agree nor disagree; (4) Agree; (5) Strongly agree. |
| --- | --- |
| An indicator is a measurement that reflects health characteristics in a given population | 5-point Likert Scale:(1) Strongly disagree; (2) Disagree; (3) Neither agree nor disagree; (4) Agree; (5) Strongly agree. |
| Indicators are dynamic, reflecting specific time-linked | 5-point Likert Scale:(1) Strongly disagree; (2) Disagree; (3) Neither agree nor disagree; (4) Agree; (5) Strongly agree. |
| Health indicators can be used to describe disease burden in a specific population group | 5-point Likert Scale:(1) Strongly disagree; (2) Disagree; (3) Neither agree nor disagree; (4) Agree; (5) Strongly agree. |
| Health indicators can be used to forecast the risk of disease outbreaks and helping to prevent epidemic/pandemic | 5-point Likert Scale:(1) Strongly disagree; (2) Disagree; (3) Neither agree nor disagree; (4) Agree; (5) Strongly agree. |
| Indicators are used in public health to drive decision-making for the health of the community | 5-point Likert Scale:(1) Strongly disagree; (2) Disagree; (3) Neither agree nor disagree; (4) Agree; (5) Strongly agree. |
| Regular monitoring indicators can provide feedback to improve decision-making in healthcare systems | 5-point Likert Scale:(1) Strongly disagree; (2) Disagree; (3) Neither agree nor disagree; (4) Agree; (5) Strongly agree. |
| Health indicators should have a common and clear definition for all Primary Care Providers | 5-point Likert Scale:(1) Strongly disagree; (2) Disagree; (3) Neither agree nor disagree; (4) Agree; (5) Strongly agree. |
| Health indicators should be reproducible | 5-point Likert Scale:(1) Strongly disagree; (2) Disagree; (3) Neither agree nor disagree; (4) Agree; (5) Strongly agree. |
| Health Indicators should be feasible so that tend to be automatic extraction from database | 5-point Likert Scale:(1) Strongly disagree; (2) Disagree; (3) Neither agree nor disagree; (4) Agree; (5) Strongly agree. |

**SECTION 4 POPULATION DISAGREGATION LEVEL OF INDICATORS**

| COVID-19 primary care indicators should be disaggregated by sex | 5-point Likert Scale:(1) Strongly disagree; (2) Disagree; (3) Neither agree nor disagree; (4) Agree; (5) Strongly agree. |
| --- | --- |
| COVID-19 primary care indicators should be disaggregated by age | 5-point Likert Scale:(1) Strongly disagree; (2) Disagree; (3) Neither agree nor disagree; (4) Agree; (5) Strongly agree. |
| COVID-19 primary care indicators should be disaggregated by group ethnic and/or migrant situation | 5-point Likert Scale:(1) Strongly disagree; (2) Disagree; (3) Neither agree nor disagree; (4) Agree; (5) Strongly agree. |
| COVID-19 primary care indicators should be disaggregated by vulnerable populations (low socioeconomic status, health illiteracy, homeless people, etc.) | 5-point Likert Scale:(1) Strongly disagree; (2) Disagree; (3) Neither agree nor disagree; (4) Agree; (5) Strongly agree. |
| Would you like to add other indicator regarding section 4? | Free text |

SECTIONS 5-11

We are going to describe indicators in section 5 to 11. All these indicators have been used in Europe to describe the role of primary care in the pandemic. The sections are divided in the different areas where primary care was involved in providing care to suspicious COVID-19 cases or to COVID-19 cases. In all the next sections, first, you have to rate the indicator in a 5 Likert Scale to describe the usefulness of each indicator in describing the activity of COVID-19 in primary care. Once, you have rated the indicator, you would have to say if the rated indicator can be useful in a practice/health centre level or in a national/regional level

**SECTION 5 INDICATORS REGARDING ROLE CENTINEL NETWORK**

How do you find the usefulness of the following indicators to monitor the pandemic in primary care?

| **Positive cases for SARS-CoV-2 (COVID-19) seen by the Sentinelles network**  Definition: Numerator: Positive cases for SARS-CoV-2 (COVID-19) seen by the Sentinelles network Denominator: Total population of a country or region | 5-point Likert Scale:(1) Not so useful; (2) Not at all useful; (3) Somewhat useful;(4) Very useful  ; (5) Extremely useful |
| --- | --- |
| Which level of disaggregation would you choose to "Positive cases for SARS-CoV-2 (COVID-19) seen by the Sentinelles network"? | Practice/Health centre; Regional/National; Both levels |
| **Positivity rates to SARS-CoV-2 (COVID-19) among all the respiratory infections by the Sentinelles network**  Definition: Numerator: Positivity rates to SARS-CoV-2 (COVID-19) among all the respiratory infections by the Sentinelles network Denominator: Total population of a country or region | 5-point Likert Scale:(1) Not so useful; (2) Not at all useful; (3) Somewhat useful;(4) Very useful  ; (5) Extremely useful |
| Which level of disaggregation would you choose to "Positivity rates to SARS-CoV-2 (COVID-19) among all the respiratory infections by the Sentinelles network"? | Practice/Health centre; Regional/National; Both levels |
| **Estimated incidence of COVID-19 cases per 100,000 population with respiratory signs observed in general practice through the Sentinelles network**  Definition: Numerator: Number of Covid19 cases with respiratory signs from Sentinelles network. Denominator. region or country's total population | 5-point Likert Scale:(1) Not so useful; (2) Not at all useful; (3) Somewhat useful;(4) Very useful  ; (5) Extremely useful |
| Which level of disaggregation would you choose to "Positivity rates to SARS-CoV-2 (COVID-19) among all the respiratory infections by the Sentinelles network"? | Practice/Health centre; Regional/National; Both levels |
| How often would you publish the indicators of section 5? | Daily; Weekly; Monthly |
| Would you like to add other indicator or comment regarding section 5? | Free text |

**Section 6: INDICATORS REGARDING SUSPICIOUS COVID-19 CASES**

How do you find the usefulness of the following indicators to monitor the pandemic in primary care?

| **Percentage of cases of COVID-19 among all respiratory infection cases in PHC**  Definition: Numerator: Number of cases of COVID-19 in primary care, region or country. Denominator: Number of all respiratory infection cases in primary care, region or country | 5-point Likert Scale:(1) Not so useful; (2) Not at all useful; (3) Somewhat useful;(4) Very useful  ; (5) Extremely useful |
| --- | --- |
| Which level of disaggregation would you choose to "Percentage of cases of COVID-19 among all respiratory infection cases in PHC"? | Practice/Health centre; Regional/National; Both levels |
| **Total COVID-19 cases with positive test in primary care**  Definition: Numerator: Number of primary care COVID-19 cases with positive test in practice, region or country. Denominator: Total Primary care COVID- 19 Tests performed in practice, region or country | 5-point Likert Scale:(1) Not so useful; (2) Not at all useful; (3) Somewhat useful;(4) Very useful  ; (5) Extremely useful |
| Which level of disaggregation would you chose to "Total COVID-19 cases with positive test in primary care"? | Practice/Health centre; Regional/National; Both levels |
| How often would you publish the indicators of section 6? | Daily; Weekly; Monthly |
| Would you like to add other indicator or comment regarding section 6? | Free text |

**Section 7 INDICATORS REGARDING PRIMARY CARE FOLLOW-UP TO COVID-19 PATIENTS**

How do you find the usefulness of the following indicators to monitor the pandemic in primary care?

| **Number of COVID-19 patients who were follow-up in primary care (nurse and/or GP) for all reasons**  Definition: Numerator: Number of COVID-19 patients who were follow-up in primary care in a period. Denominator: total number of patients visited in primary care in a period | 5-point Likert Scale:(1) Not so useful; (2) Not at all useful; (3) Somewhat useful;(4) Very useful  ; (5) Extremely useful |
| --- | --- |
| Which level of disaggregation would you choose to "Number of COVID-19 patients who were follow-up in primary care (nurse and/or GP) for all reasons"? | Practice/Health centre; Regional/National; Both levels |
| **Primary care follow-up ratio (nurses and/or GP): follow-up ratio of cases and contacts**  Definition: Numerator: Family medicine follow-up COVID-19 cases Denominator: Family medicine follow-up COVID-19 contacts | 5-point Likert Scale:(1) Not so useful; (2) Not at all useful; (3) Somewhat useful;(4) Very useful  ; (5) Extremely useful |
| Which level of disaggregation would you choose to "Primary care follow-up ratio (nurses and/or GP): follow-up ratio of cases and contacts"? | Practice/Health centre; Regional/National; Both levels |
| How often would you publish the indicators of section 7? | Daily; Weekly; Monthly |
| Would you like to add other indicator or comment regarding section 7? | Free text |

**Section 8 INDICATORS REGARDING THE FOLLOW-UP OF PRIMARY CARE NURSES TO COVID-19 PATIENTS**

How do you find the usefulness of the following indicators to monitor the pandemic in primary care?

| **Number of any contacts with nurse with COVID-19 recorded as reason for the contact**  Definition: Numerator: Number of any contacts with nurse with COVID-19 recorded as reason for the contact Denominator: total contacts with nurse in a period | 5-point Likert Scale:(1) Not so useful; (2) Not at all useful; (3) Somewhat useful;(4) Very useful  ; (5) Extremely useful |
| --- | --- |
| Which level of disaggregation would you choose to "Number of any contacts with nurse with COVID-19 recorded as reason for the contact"? | Practice/Health centre; Regional/National; Both levels |
| **Number of nurse home visits with COVID-19 recorded as reason for home care**  Definition: Numerator: Number of nurse home visits with COVID-19 recorded as reason for home care Denominator: total nurse home visits with nurse in a period | 5-point Likert Scale:(1) Not so useful; (2) Not at all useful; (3) Somewhat useful;(4) Very useful  ; (5) Extremely useful |
| Which level of disaggregation would you choose to "Number of nurse home visits with COVID-19 recorded as reason for home care "? | Practice/Health centre; Regional/National; Both levels |
| **Number of nurse telephone consultations with COVID-19 recorded as reason for consultation**  Definition: Numerator: Number of nurse telephone consultations with COVID-19 recorded as reason for consultation Denominator: total telephone contacts with nurse in a period | 5-point Likert Scale:(1) Not so useful; (2) Not at all useful; (3) Somewhat useful;(4) Very useful  ; (5) Extremely useful |
| Which level of disaggregation would you choose to "Number of nurse telephone consultations with COVID-19 recorded as reason for consultation "? | Practice/Health centre; Regional/National; Both levels |
| **Number of nurse control home visit with COVID-19 recorded as reason for home visit**  Definition: Numerator: Number of nurse control home visit with COVID-19 recorded as reason for home visit Denominator: total home visits with nurse in a period | 5-point Likert Scale:(1) Not so useful; (2) Not at all useful; (3) Somewhat useful;(4) Very useful  ; (5) Extremely useful |
| Which level of disaggregation would you choose to "Number of nurse control home visit with COVID-19 recorded as reason for home visit"? | Practice/Health centre; Regional/National; Both levels |
| How often would you publish the indicators of section 8? | Daily; Weekly; Monthly |
| Would you like to add other indicator or comment regarding section 8? | Free text |

**Section 9 INDICATORS REGARDING THE FOLLOW-UP OF COVID-19 PATIENTS IN PRIMARY CARE**

How do you find the usefulness of the following indicators to monitor the pandemic in primary care?

| **Number of phone consultations to patients with COVID-19 or patients close family member (by GP)**  Definition, Numerator: Number of phone consultations to patients with COVID-19 or patients close family member by GP Denominator: Total number of phone consultations to patients by GP | 5-point Likert Scale:(1) Not so useful; (2) Not at all useful; (3) Somewhat useful;(4) Very useful  ; (5) Extremely useful |
| --- | --- |
| Which level of disaggregation would you choose to "Number of phone consultations to patients with COVID-19 or patients close family member (by GP)"? | Practice/Health centre; Regional/National; Both levels |
| **Number of email consultations to patients with COVID-19 or patients close family member (by GP)**  Definition, Numerator: Number of email consultations to patients with COVID-19 or patients close family member (by physician) Denominator: Total number of email consultations to patients by GP | 5-point Likert Scale:(1) Not so useful; (2) Not at all useful; (3) Somewhat useful;(4) Very useful  ; (5) Extremely useful |
| Which level of disaggregation would you choose to "Number of email consultations to patients with COVID-19 or patients close family member (by GP) "? | Practice/Health centre; Regional/National; Both levels |
| **Number of face-to-face visits to GP with COVID-19 recorded as reason for the visit**  Definition, Numerator: Number of face-to-face visits to GP with COVID-19 recorded as reason for the visit. Denominator: Total number of face-to-face consultations to patients by GP | 5-point Likert Scale:(1) Not so useful; (2) Not at all useful; (3) Somewhat useful;(4) Very useful  ; (5) Extremely useful |
| Which level of disaggregation would you choose to "Number of face-to-face visits to GP with COVID-19 recorded as reason for the visit"? | Practice/Health centre; Regional/National; Both levels |
| **Number of first visits (examinations) with COVID-19 recorded as reason for the visit (by GP)**  Definition, Numerator: Number of first visits (examinations) with COVID-19 recorded as reason for the visit (by GP) Denominator: Total number of first visits to patients by GP | 5-point Likert Scale:(1) Not so useful; (2) Not at all useful; (3) Somewhat useful;(4) Very useful  ; (5) Extremely useful |
| Which level of disaggregation would you choose to "Number of first visits (examinations) with COVID-19 recorded as reason for the visit (by GP) "? | Practice/Health centre; Regional/National; Both levels |
| **Number of control visits (examinations) with COVID-19 recorded as reason for the visit (by GP)**  Definition, Numerator: Number of control visits (examinations) with COVID-19 recorded as reason for the visit (by GP) Denominator: Total number of control visits to patients by GP | 5-point Likert Scale:(1) Not so useful; (2) Not at all useful; (3) Somewhat useful;(4) Very useful  ; (5) Extremely useful |
| Which level of disaggregation would you choose to "Number of control visits (examinations) with COVID-19 recorded as reason for the visit (by GP) "? | Practice/Health centre; Regional/National; Both levels |
| **Number of first home visits with COVID-19 recorded as reason for home visit (by GP)**  Definition, Numerator: Number of first home visits with COVID-19 recorded as reason for home visit (by GP) Denominator: Total number of first home visits to patients by GP | 5-point Likert Scale:(1) Not so useful; (2) Not at all useful; (3) Somewhat useful;(4) Very useful  ; (5) Extremely useful |
| Which level of disaggregation would you choose to "Number of first home visits with COVID-19 recorded as reason for home visit (by GP) "? | Practice/Health centre; Regional/National; Both levels |
| **Number of control home visits with COVID-19 recorded as reason for home visit (by GP)**  Definition, Numerator: Number of control home visits with COVID-19 recorded as reason for home visit (by physician) Denominator: Total number of control home visits to patients by GP | 5-point Likert Scale:(1) Not so useful; (2) Not at all useful; (3) Somewhat useful;(4) Very useful  ; (5) Extremely useful |
| Which level of disaggregation would you choose to "Number of control home visits with COVID-19 recorded as reason for home visit (by GP) "? | Practice/Health centre; Regional/National; Both levels |
| How often would you publish the indicators of section 9? | Daily; Weekly; Monthly |
| Would you like to add other indicator or comment regarding section 9? |  |

**Section 10: INDICATORS REGARDING NUMBER OF PROCEDURES IN PHC TO COVID-19 PATIENTS**

How do you find the usefulness of the following indicators to monitor the pandemic in primary care?

| **Total number of procedures to patients in primary care with COVID-19 recorded as reason for procedures**  Definition: Numerator: Total number of procedures to patients in primary care with COVID-19 recorded as reason for procedures Denominator: Total number of procedures to all patients in the practice in a period | 5-point Likert Scale:(1) Not so useful; (2) Not at all useful; (3) Somewhat useful;(4) Very useful  ; (5) Extremely useful |
| --- | --- |
| Which level of disaggregation would you chose to "Total number of procedures to patients in primary care with COVID-19 recorded as reason for procedures"? | Practice/Health centre; Regional/National; Both levels |
| **Number of COVID-19 patients who were examined in PHC (X-ray or/and phlebotomy)**  Definition: Numerator: Number of COVID-19 patients who were examined in primary care (X-ray or/and phlebotomy) Denominator: Total number of patients examined in PHC (X ray or/and phlebotomy) by GP | 5-point Likert Scale:(1) Not so useful; (2) Not at all useful; (3) Somewhat useful;(4) Very useful  ; (5) Extremely useful |
| Which level of disaggregation would you chose to "Number of COVID-19 patients who were examined in primary care (X-ray or/and phlebotomy) "? | Practice/Health centre; Regional/National; Both levels |
| How often would you publish the indicators of section 10? | Daily; Weekly; Monthly |
| Would you like to add other indicator or comment regarding section 10? | Free text |

**Section 11 IINDICATORS REGARDING NUMBER OF PROCEDURES IN PHC TO COVID-19 PATIENTS**

How do you find the usefulness of the following indicators to monitor the pandemic in primary care?

| **Sick leaves processed by GPs of patients in COVID-19 quarantine**  Definition: Numerator: Sick leaves processed by GPs of patients in COVID-19 quarantine. Denominator: Total of sick leaves by GPs in a period | 5-point Likert Scale:(1) Not so useful; (2) Not at all useful; (3) Somewhat useful;(4) Very useful  ; (5) Extremely useful |
| --- | --- |
| Which level of disaggregation would you chose to “Sick leaves processed by GPs of patients in COVID-19 quarantine “? | Practice/Health centre; Regional/National; Both levels |
| **Sick leaves processed by GPs of COVID-19 patients in isolation**  Definition: Numerator: Sick leaves processed by GPs of COVID-19 patients in isolation. Denominator: Total of sick leaves by GPs in a period | 5-point Likert Scale:(1) Not so useful; (2) Not at all useful; (3) Somewhat useful;(4) Very useful  ; (5) Extremely useful |
| Which level of disaggregation would you chose to "Sick leaves processed by GPs of COVID-19 patients in isolation"? | Practice/Health centre; Regional/National; Both levels |
| How often would you publish the indicators of section 11? | Daily; Weekly; Monthly |
| Would you like to add other indicator or comment regarding section 11? | Free text |

**Thank you very much for finishing the first Delphi round, you will receive the second round in 4-8 weeks.**

**Supplement 4: Information for the participant**

**INFORMATION FOR THE PARTICIPANT**

**Title:** Description of Primary Care data during COVID-19 in Europe: descriptive study

**Principal Investigator, service / unit and center**: Marina Guisado Clavero and Sara Ares Blanco, Health Technician of the Multiprofessional Teaching Unit of Family and Community Care of the North area and General practitioner of Federica Montseny Health centre of ​​the Community of Madrid

**Promoter / funder (if applicable)**: not applicable

**Version number and date:** version 3, 11/8/2021

**Introduction**

We are writing you to inform about a study in which you are invited to participate. Our intention is that you receive correct and sufficient information so that you can evaluate and judge whether or not you want to participate in this study. We will clarify any doubts that may arise at any time. In addition, you can consult with the people you deem appropriate.

**Voluntary participation**

You should know that your participation is voluntary and that you can decide not to participate or change your decision and withdraw your consent at any time, without thereby altering your relationship with your doctor or causing any harm to your treatment.

**Description and general objective of the study**

We are conducting a study in the field of primary care that aims to obtain available information on COVID-19 patients at the primary care level of care in the countries that make up the European Union, the common economic area and the OCDE. Obtaining this information allows us to know the accessibility and transparency of healthcare data, in order to establish new strategies in future pandemics and policies aimed at European citizens. You are a primary care physician and you can collaborate in the description of the healthcare dynamics that have occurred during COVID-19 in your country. For this reason, we ask you to participate voluntarily in this project that involves the participation of multiple primary care professionals from various European countries.

Risks and annoyances derived from your participation in the study. Possible benefits

You may not get any health benefits from participating in this study.

Participation in this study carries no risk to you. The data will be treated anonymously. The benefits that can come from this project are aimed at building new European policies on primary care.

**Economic compensation**

Your participation in the study will not entail any additional expense nor will you have any financial compensation.

**Contact**

If you have any questions in the future about the disclosure or use that may be made of your information, if you have questions, concerns or complaints about the study or your participation in it, you should contact:

• Dra Marina Guisado Clavero

[marina.guisado@salud.madrid.org](mailto:marina.guisado@salud.madrid.org)

• Dra Sara Ares Blanco

[sara.ares@salud.madrid.org](mailto:sara.ares@salud.madrid.org)

**Supplement 5:** Informed consent for Delphi participants ( project 3)

**INFORMATION FOR THE PARTICIPANT**

**Eurodata, Description of Primary Care data during COVID-19 in Europe, project 3: Delphi study**

Dear colleague:

We would like to invite you to take part in a Delphi consensus study. Before you decide whether or not you would like to take part, it is important for you to consider why the research is being done and what it will involve. Please read this information sheet carefully.

**Description and general objective of the study**

The Eurodata project aims to obtain available information on how primary care was organized during the COVID-19 pandemic in Europe. We have studied any health indicators regarding COVID-19 activity in primary health care (PHC) in 30 European countries. We found that only 13 countries recorded information regarding the role of PHC. The main indicators in PHC were: total number of cases in PHC, number of follow-ups in PHC, General Practitioner’s (GP) COVID-19 tests referrals, proportion of COVID-19 cases among respiratory illnesses consultations, sick leaves issued by GPs, examination and complementary tests. There was a high heterogeneity of indicators.

This leads us to believe that **it is necessary to make an agreement of a common set of primary care COVID-19 indicators in Europe among GPs and Public Health officers.** At least, **two GPs and two Public Health officer will be invited from each participating country**. Obtaining this information would allow us to know the accessibility and transparency of healthcare data, in order to establish new strategies in future pandemics and policies aimed at European citizens.

**Why have I been invited to take part?**

As an established GP or Public Health officer we are keen to gain your views about which indicators may be better in describing the activity in PHC during the pandemic**. This would involve completing a brief online questionnaire, rating possible PHC indicators.** **It is envisaged that this should take approximately 15-20 minutes**. You would subsequently receive a reminder of your ratings, a summary of the group’s responses and a further online questionnaire to re-rate the original list of predictors. This process would continue **until a group consensus is achieved in two Delphi rounds** or in a third Delphi round if consensus is not reached before. In order to allow timely conclusion of the study we would respectfully request a response time of 1 month for completion of each round. The questionnaire will be sent by an e-mail from the Universidad Miguel Hernández de Elche ( Spain).

**Who is organizing and funding the research?**

The Delphi study will be conducted by Dr. Pilar Astier-Peña (Technical Advisor for Quality and Safety. Territorial Healthcare Quality Unit. Territorial Healthcare Direction of Camp de Tarragona. Healthcare Institute of Catalonia. Health Departament. Generalitat de Catalunya, Spain) as the main researcher with the support of Raquel Gómez Bravo, Marina Guisado-Clavero and Sara Ares-Blanco. The Eurodata project has received a grant from the European General Practice Research Network (EGPRN).

**Confidentiality and Data Protection**

No personal information will be collected and survey responses will be collated anonymously using an identifying number known only to the participant and lead investigator. All responses received in the study will be strictly confidential, and your identity will not be divulged. Direct quotes to free-text answers may be used as part of the study report or later Delphi iterations, but these will be not be traceable back to you.

Survey responses will be collected online using an online software from the University of Alicante (Spain). Data will be stored only for the duration of the research project in the University of Alicante secure server and then deleted. You have the right to access submitted information according to the Spanish data protection laws.

**Research ethics**

The ethical approval was obtained from the Ethics Committee of the Hospital Universitario La Paz (Madrid, Spain), ID PI-5030. A copy of the ethics committee approval is available on request.

**Economic/Academic compensation**

Your participation in the study will not entail any additional expense nor will you have any financial compensation. We are offering being part of a collaborative authorship in a paper and/or in a presentation in a WONCA (World Family Doctors) conference network if you complete all the Delphi rounds.

If you are interested in being part of the collaborative authorship, you should answer a question in round 2 to give consent that the research team contact you to share the draft of the study/abstract and to provide your affiliation.

**What do I do now?**

Thank you for reading this information sheet and for considering taking part in this research. Please let us know whether or not you would like to take part by replying to this email or to the person who shared this study with you. If you have any questions or concerns, please do not hesitate to contact us.

Dr. Sara Ares Blanco [sararesb@gmail.com](mailto:sararesb@gmail.com) (GP in Federica Montseny Health center, Madrid, Spain).

Dr. Raquel Gómez Bravo [raquelgomezbravo@gmail.com](mailto:raquelgomezbravo@gmail.com) (Postdoctoral researcher at CHNP, Rehaklinik. Ettelbruck. Luxembourg)
